# Modeling the change trajectory of sleep duration and its associated factors: based on an 11-year longitudinal survey in China

**DOI:** 10.1186/s12889-021-12017-8

**Published:** 2021-10-30

**Authors:** Junyan Fang, Zhonglin Wen, Jinying Ouyang, Huihui Wang

**Affiliations:** 1 School of Psychology and Center for Studies of Psychological Application, South China Normal University, Tianhe District, Guangzhou City, Guangzhou province, 510631 China; 2 Key Laboratory of Brain, Cognition and Education Sciences (SCNU), Ministry of Education, Guangzhou, China

**Keywords:** Sleep duration, Longitudinal study, Trajectory, Associated factors

## Abstract

**Background:**

Sleep duration is a vital public health topic, yet most existing studies have been limited to cross-sectional surveys or inconsistent classifications of sleep duration categories, and few characterized its continuous development process. The current study aimed to depict its change trajectory in the general population and identify associated factors from a dynamic perspective.

**Methods:**

A total of 3788 subjects (45.4% male, mean age 46.72 ± 14.89 years) from the China Health and Nutrition Survey were recruited, and their daily sleep duration for five consecutive measurements from 2004 to 2015 was recorded. We adopted latent growth modelling to establish systematic relations between sleep duration and time. Participants’ sociodemographic characteristics, lifestyle, and health factors were taken as covariates.

**Results:**

The change in sleep duration could be depicted by a linear decreasing trajectory with the mean yearly decrease at 2.5 min/day. The trajectory did not differ by residence, BMI category, chronic disease situation, smoking status, or drinking status. Moreover, there were sex and age differences in the trajectory, and females and those under 30 were prone to larger decrease rates.

**Conclusion:**

The quantified yearly change in sleep duration provided insights for the prediction and early warning of insufficient sleep. Public health interventions focusing on slowing down the decrease rates among females and young individuals are warranted.

## Background

The importance of sleep in terms of duration is increasingly coming under scrutiny. Sleep duration varies across the lifespan [1], and researchers have devoted considerable attention to its change [2, 3]. In most empirical investigations, sleep duration was found to show a decreasing tendency, and this result was verified by the increase in the proportion of insufficient sleep [4–7]. For example, in a survey of high school students, the prevalence of insufficient sleep (< 8 h/day) increased by 45.9% in a year [6]. A longitudinal study found that the average daily sleep duration among Spanish children decreased by 0.8 min/day per year [2]. A systematic review of studies from 1905 to 2008 of children from 20 countries reported a decline of 0.75 min/day per year [8].

Evidence from observational research has indicated that there are several influential factors of sleep duration, such as sex, age, geographical region, body mass index (BMI) category, and hypertension [2, 9, 10]. Smoking and alcohol consumption were also found to be related to short sleep duration [10–12].

However, most existing studies have utilized varying definitions of insufficient/short sleep, and different cut-off values were adopted, such as 6 h/day [3], 7 h/day [13, 14], and 8 h/day [5, 6]. In other instances, researchers used the self-assessed status of insufficient sleep rather than objective indices [7]. Inconsistency in the classification of sleep duration category made it a barrier for the comparison among different results, and it was also difficult to generalize previous findings.

Moreover, existing studies mostly used cross-sectional surveys, and few have paid attention to the continuous development process of sleep duration. In the limited amount of longitudinal research that tried to characterize the change in sleep duration, special participants were used, such as infancy [15], preschool children [16], and middle school students [2, 8, 17], which was inadequate to obtain a whole picture of this constantly changing variable across the lifespan.

All these factors necessitate a longitudinal examination of the change in sleep duration in a large and representative population so that we can comprehensively describe its trajectory over time. To formulate the systematic relationship between sleep duration and time, we analysed a five-wave longitudinal dataset of representative participants from the China Health and Nutrition Survey (CHNS). Latent growth modelling (LGM) was utilized to picture the changing pattern and to further explore influential factors of this dynamic process.

## Methods

### Study subjects

Data for this study was obtained from the CHNS. The CHNS is an ongoing longitudinal survey in China, which is conducted jointly by the Carolina Population Center at the University of North Carolina at Chapel Hill (CPC-UNC-Chapel Hill) and the National Institute for Nutrition and Health at the Chinese Center for Disease Control and Prevention (NINH-CCDCP). It comprises a total of ten waves (1989, 1991, 1993, 1997, 2000, 2004, 2006, 2009, 2011, and 2015). The CHNS employed a multistage, random cluster process to draw a sample of about 7200 households with over 30,000 individuals in 15 provinces, which represented approximately 50% of the Chinese population. The investigation instruments and process for this survey were approved by the Institutional Review Committees of CPC- UNC-Chapel Hill and NINH-CCDCP. All participants provided written informed consent. Details about the study design are available elsewhere [13, 14, 18].

**Fig. 1:**
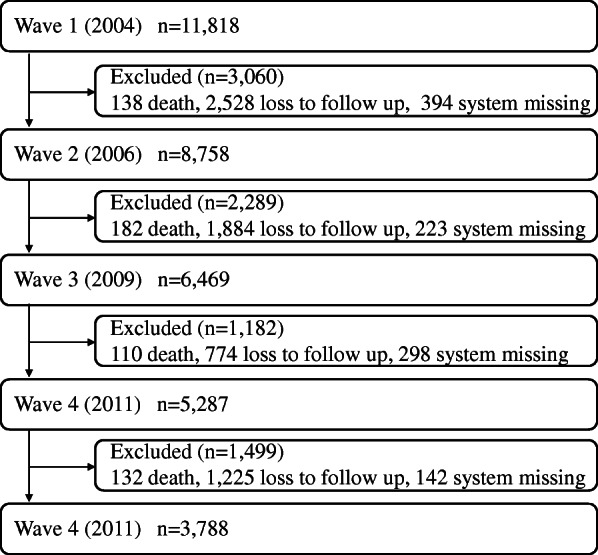
Flow chart illustrating the sample selection for the present study. Note: System missing, failed to report on sleep duration

In the CHNS, measurements of sleep duration started in 2004, so we used surveys from 2004 to 2015 that constituted a five-wave dataset. The original sample of 11,818 individuals was interviewed in 2004 (T1), and participants were excluded if they died, were lost to follow-up, or failed to respond to the focal variable. The attrition rates at each measurement were 25.89% (T2), 26.14% (T3), 18.27% (T4), and 28.35% (T5). Attrition is almost inevitable in longitudinal studies, especially given the long timeline for follow-up. Finally, a total of 3788 eligible participants were analysed (see Fig. 1).

The sample analysed here had a similar sex ratio (45.4% male) to those participants who were excluded (50.7% male), and the proportions of people living in rural areas were also very similar (69.9, 64.6%). Our sample was slightly older (mean age at T1 was 46.72) than the excluded individuals (mean age at T1 was 37.74). In addition, the sample we used reported a shorter average sleep duration at baseline than excluded participants (*M*_used_ = 8.24, *M*_excluded_ = 8.52, *p* < 0.01, Cohen’s *d* = 0.18).

### Measures

#### Sleep duration

Sleep duration was assessed by the self-report questionnaire, which asked participants the following question: “How many hours each day do you usually sleep, including during both daytime and night-time?”. Self-reported measures of sleep duration were recommended in both clinical and population-based studies [7, 13, 19].

#### Covariates

Relevant covariates were collected through questionnaires administered by trained investigators. Sociodemographic factors were sex (male/female), residence (urban/rural), age and BMI. BMI values were calculated from measured height and weight, and the miss rates of height and weight were rather high in subsequent surveys, so we only analysed the initial BMI category. Lifestyle factors were smoking status (not smoking/smoking) and alcohol drinking status (not drinking/drinking). The health-related factor was chronic disease situation (never diagnosed with hypertension, diabetes, myocardial infarction, apoplexy, or cancer/diagnosed with at least one). Sex, residence, age (categorized into three classes: < 30/30 ~ 50/> 50 years old at T1), initial BMI (classified into three classes: < 18.5/18.5 ~ 24/24 kg/m^2^), and chronic disease situation were considered as time-invariant covariates. Smoking and drinking status were regarded as time-variant covariates.

#### Analytic approach

The LGM is a powerful tool to infer an underlying growth process [20]. The standard LGM takes on a linear trajectory, in which the development trajectory is denoted by two latent factors, the intercept factor (*I*), which indicates the initial level of the variable, and the slope factor (*S*), which describes the variation per time unit. The LGM is expressed by the following equation, in which *i* denotes each individual and *t* denotes the measured time.

1 $$ {Sleep\ duration}_i={I}_i-\left(t-1\right)\ast {S}_i $$

For the current study, we used the default linear trajectory to depict the change in sleep duration. A nonlinear assumption was also possible, but for a given construct, there was little difference between linear and nonlinear results [17]. For the sake of interpretability, the linear LGM was adopted in current analyses. Considering that measurements spanned unevenly (T1 = 2004, T2 = 2006, T3 = 2009, T4 = 2011, T5 = 2015), we set the loadings on the slope factor to be equal to 0, 2, 5, 7, 11 for each cohort (reflecting the passage of time) so that the time unit would be a year and we could grasp the change year by year.

**Fig. 2:**
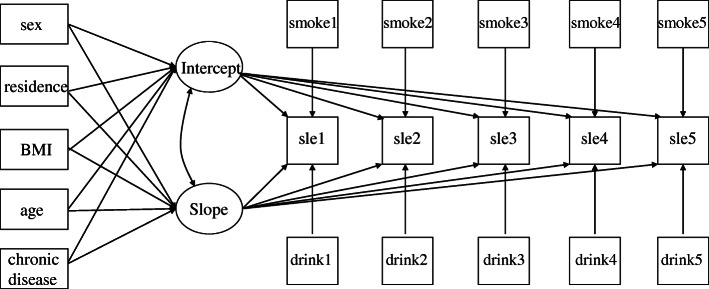
Conditional LGM of sleep duration for five-time points. Note: sle, sleep duration. BMI, initial BMI category; smoke, smoking status; drink, alcohol drinking status

Three stages of LGM analyses were used here. Stage 1, unconditional LGM. We conducted an overall LGM analysis to reveal the general change trajectory of sleep duration. Stage 2, conditional LGM. A conditional model was analysed to identify influential factors, and both time-invariant and time-varying covariates were considered (see Fig. 2). Stage 3, subgroup LGM. Stratification analyses were conducted to further illustrate the effects of covariates.

We used SPSS 24.0 [21] and Mplus 8.0 [22] to conduct the analyses. In LGM analyses, the missing data of covariates were handled by the full information maximum likelihood (FIML) estimator, a popular technique to deal with missing data based on available information. Several indices were used to assess the model fit, such as the χ^2^ statistic, comparative fit index (CFI), Tucker-Lewis index (TLI), and root-mean-square error of approximation (RMSEA) [23].

## Result

### Descriptive analysis

The sample for the current analysis was made up of individuals with valid responses to sleep duration through all five measurements (*n* = 3788). Table 1 shows the statistics of key variables at different measurements.

**Table 1:** Descriptive characters of the sample at five waves

	2004 (T1)	2006 (T2)	2009 (T3)	2011 (T4)	2015 (T5)
***Sample size***	3788	–	–	–	–
***Focal variable***					
Sleep duration	8.24 ± 1.34	8.15 ± 1.28	7.97 ± 1.29	7.87 ± 1.26	7.81 ± 1.28
< 6 h/day, n(%)	254 (6.7%)	293 (7.7%)	390 (10.3%)	455 (12.0%)	483 (12.8%)
6 ~ 7 h/day, n(%)	639 (16.9%)	666 (17.6%)	706 (18.6%)	753 (19.9%)	769 (20.3%)
7 ~ 8 h/day, n(%)	1655 (43.7%)	1662 (43.9%)	1771 (46.8%)	1709 (45.1%)	1777 (46.9%)
≥ 8 h/day, n(%)	1240 (32.7%)	1167 (30.8%)	921 (24.3%)	871 (23.0%)	759 (20.0%)
***Time-invariant covariates***					
Male, n(%)	1718 (45.4%)	–	–	–	–
Urban residence, n(%)	1132 (29.9%)	–	–	–	–
Age (T1)	46.72 ± 14.89	–	–	–	–
< 30 years old, n(%)	357 (9.4%)	–	–	–	–
30 ~ 50 years old, n(%)	1837 (48.5%)	–	–	–	–
≥ 50 years old, n(%)	1594 (42.1%)	–	–	–	–
BMI (T1)	23.02 ± 3.59	–	–	–	–
< 18.5 kg/m^2^, n(%)	287 (8.1%)	–	–	–	–
18.5 ~ 24 kg/m^2^, n(%)	1928 (63.1%)	–	–	–	–
≥ 24 kg/m^2^, n(%)	1315 (36.9%)	–	–	–	–
Chronic diseases, n(%)	842 (22.2%)	–	–	–	–
***Time-varying covariates***					
Smoking, n(%)	1153 (30.4%)	1136 (30.0%)	1115 (29.4%)	1099 (29.0%)	943 (24.9%)
Alcohol-drinking, n(%)	1211 (32.0%)	1207 (31.9%)	1218 (32.2%)	1196 (31.6%)	972 (25.7%)

T, time; *n*, sample size; *M*, mean; *SD*, standard deviation

**Fig. 3:**
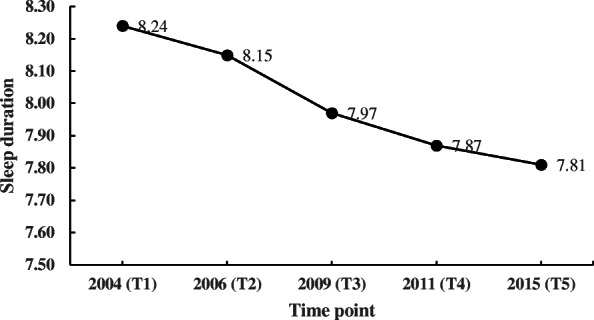
Average sleep duration at different time points

As shown in Table 1, the average sleep duration of the population presented a downward trend (also see Fig. 3). In addition, the proportion of individuals who slept less than 6 or 7 h/day increased as time went by, while the proportion of people sleeping more than 8 h/day decreased. This finding was consistent with previous research that reported the universal phenomenon of decreasing sleep duration [2–5, 9].

Sex was relatively balanced in our sample, with approximately 45.4% being males. The mean baseline age was 46.72 years old, with a standard deviation of 14.89 years old. The majority of participants were rural dwellers (69.9%), and their residence remained unchanged during the survey. The sample was made up of responders with different initial BMI categories. At baseline, a small portion (8.1%) were underweight, and some (36.9%) were overweight. Approximately 22.2% of the sample was diagnosed with at least one chronic disease. In addition, smoking and alcohol-drinking habits were also recorded, and the proportions of smokers and drinkers both decreased during the period.

### Unconditional LGM analysis

The unconditional LGM fit well with the data (*χ*^2^ = 49.68, *df* = 14, CFI = 0.96, TLI = 0.97, RMSEA = 0.03). The general mean sleep duration was 8.215 h/day (*p* < 0.001) at the initial time point, and then, it was expected to decrease by 0.041 h/day (*p* < 0.001) each year, which meant that from one year to the next, sleep duration was expected to decrease by approximately 2.5 min/day (see Table 2).

**Table 2:** Results of the unconditional LGM analysis

	Mean (***S***.***E***.)	***p***	Variance (***S***.***E***.)	***p***
***I***	8.215 (0.018)	0.000	0.518 (0.045)	0.000
***S***	−0.041 (0.002)	0.000	0.004 (0.001)	0.000
***r***	−0.630 (0.042)	0.000	–	–

*I*, intercept factor indicating the initial level; *S*, slope factor indicating the change rate;

*r*, the correlation between *I* and *S*; *S*.*E*., standard error

The correlation between the initial level and change rate was − 0.630 (*p* < 0.001), suggesting that those with longer baseline sleep tended to experience larger decreases afterwards. Then, we conducted subsequent analyses to identify influential factors.

### Conditional LGM analysis

The conditional LGM provided a tolerable fit (*χ*^2^ = 490.10, *df* = 77, CFI = 0.85, TLI = 0.84, RMSEA = 0.04). Table 3 provides the standardized estimates of the analysis.

**Table 3:** Standardized estimations of the conditional LGM analysis

	***β***	***CI*** (95%)		***S.E.***	***p***
		lower	upper		
***Time-invariant covariates***					
Sex → *I*	0.134^*^	0.019	0.249	0.059	0.023
Sex → *S*	−0.265^***^	−0.423	− 0.108	0.080	0.001
Residence → *I*	0.002^*^	0.000	0.004	0.001	0.020
Residence → *S*	−0.003^**^	− 0.006	−0.001	0.001	0.005
Age(T1) → *I*	−0.218^***^	−0.314	− 0.123	0.049	0.000
Age(T1) → *S*	0.161^*^	0.037	0.285	0.063	0.011
BMI (T1) → *I*	0.000^***^	0.000	0.001	0.000	0.000
BMI (T1) → S	0.000	−0.001	0.000	0.000	0.145
Chronic disease → *I*	0.001	0.000	0.001	0.000	0.182
Chronic disease → *S*	0.000	−0.001	0.001	0.000	0.372
***Time-varying covariates***					
Smoking → sleep duration	0.001^***^	0.001	0.001	0.000	0.000
Alcohol-drinking → sleep duration	0.001^***^	0.000	0.001	0.000	0.000

*β*, standardized regression coefficient; *I*, intercept factor indicating the initial level; *S*, slope factor indicating the change rate. *CI*, centered interval; *S.E.*, standard error

Time-varying covariate effects were set to be stable at different waves

^*^, *p* < 0.05; ^**^, *p* < 0.01; ^***^, *p* < 0.001

For time-invariant covariates, sex and age impacted both the initial level and change rate, but their effects were in opposite directions. For sex, the effects were *β* = 0.134 (*p* < 0.05) and *β* = − 0.265 (*p* = 0.001), respectively; for age, *β* = − 0.218 (*p* < 0.001) and *β* = 0.161 (*p* < 0.05). The effects of residence place and initial BMI category were significant but very small in size, and whether being diagnosed with chronic diseases showed no significant influence.

For time-varying covariates, smoking and drinking status showed significant influences at different waves, but the effects were limited (*β* = 0.001, 0.001, *ps* < 0.001).

### Subgroup LGM analyses

The above multivariate analysis revealed that sex and age had substantial influences on the change characteristics of sleep duration; therefore, we conducted stratification analyses to further illustrate their effects.

The metric invariance was assumed here, and the loadings of the intercept and slope factors were fixed to be the same as those in the overall analysis. Good model fit indices verified the metric invariance assumption, which implied that the initial level and change rate can be meaningfully compared among subgroups. Table 4 presents the summary of stratification analyses.

**Table 4:** Summary of the estimations in subgroup LGM analyses

		***χ***^**2**^***(df)***	CFI	TLI	RMSEA	***I***(h/day)	***S***(h/day)	***S*** (min/day)
***Overall analyses***								
Whole sample		49.68 (14)	0.96	0.97	0.03	8.215^***^	−0.041^***^	−2.460^***^
***Subgroup analyses***								
Sex	female	34.60(14)	0.96	0.97	0.03	8.240^***^	−0.048^***^	−2.880^***^
	male	23.00(14)	0.98	0.98	0.02	8.185^***^	−0.033^***^	−1.980^***^
Age (T1)	< 30	30.15(14)	0.91	0.93	0.06	9.302^***^	−0.114^***^	−6.840^***^
	30 ~ 50	26.24(14)	0.95	0.97	0.02	8.092^***^	−0.034^***^	−2.040^***^
	≥50	49.23(14)	0.86	0.90	0.04	8.113^***^	−0.033^***^	−1.980^***^

T, time; *I*, intercept factor; *S*, slope factor; h, hours; min, minutes; ^***^, *p* < 0.001; ^**^, *p* < 0.01; ^*^, *p* < 0.05

The results further verified the sex and age differences in the dynamic process. Compared with males, females slept longer at first (*I*_female_ = 8.240, *I*_male_ = 8.185) and experienced a steeper decrease (*S*_female_ = − 0.048, *S*_male_ = − 0.033). Among various age groups, people under 30 years old had the longest baseline sleep, and those above 50 slept slightly longer than the middle-aged group (*I*_< 30_ = 9.302, *I*_30 ~ 50_ = 8.092, *I*_≥50_ = 8.113). In addition, the young’s sleep duration was expected to decrease much faster, while the other two age groups had similar changes (*S*_< 30_ = − 0.114, *S*_30 ~ 50_ = − 0.034, *S*_≥50_ = − 0.033).

## Discussion

To the best of our knowledge, this was the first study that adopted large, representative longitudinal samples to model the general change pattern of sleep duration and quantified its change amount over time. Overall, we found that the change in sleep duration could be depicted by a linear decreasing trajectory with the mean yearly decrease at 2.5 min/day. The trajectory did not differ by residence, BMI category, chronic disease situation, smoking status, or drinking status. Additionally, females and those under 30 were prone to larger decrease rates of sleep duration.

By formulating the systematic relationship between sleep duration and time, we identified a linear change trajectory of sleep duration, which was consistent with preceding research; for example, in a survey of Korean middle school children, it reported a linear decreasing trajectory of sleep duration [17]. Our findings verified previous results and further generalized this linear change trajectory of sleep duration into a more general population. In addition, we found that the yearly expected decrease in sleep duration was 0.041 h/day (approximately 2.5 min/day). In a survey of Spanish children, researchers found that the average daily sleep duration decreased by 0.8 min/day per year [2]. A systematic review of studies from 1905 to 2008 of children from 20 countries reported a decline of 0.75 min/day per year [8]. The findings were similar to our results, and the slight difference might be ascribed to the overall poor sleep condition in China, where the prevalence of sleep disorders exceeded the global average at 27% [24]. The quantified yearly change amount of sleep duration could help predict the future total sleep time and provide early warning of inadequate sleep. For example, the average sleep duration in 2004 was 8.215 h/day, and with the expected yearly decrease rate of 0.041 h/day, the sleep duration was predicted to fall below 7.5 h/day in 2022.

The results revealed sex and age differences during the change process of sleep duration. Regarding the effects of sex, our results indicated that compared to males, females tended to report a slightly longer sleep duration at first; however, it is noteworthy that females suffered a much faster decrease, which heralded that the difference in initial sleep duration would vanish over time and that females were more likely to suffer poor sleep in the future. A meta-analysis of 65 cross-sectional studies reported that the age-related declines in total sleep time were larger in women than in men [25]. There is considerable evidence that female sex hormones, including oestrogen and progestogen, directly impact women’s sleep [25–28]. Female bodies experienced hormone changes throughout a lifetime, and their sleep disturbances were most pronounced during periods characterized by significant hormonal change. For example, teenage girls in puberty were at nearly three times the risk of developing insomnia relative to boys [29]. In addition, females tend to report worse sleep quality and more sleep disturbances during pregnancy and menopause, which are two key periods of hormonal flux [27, 30]. Moreover, dual pressures from family and work made women more vulnerable to stress [31], and females generally exhibited a stronger stress response than males [27], which might also contribute to their fast decline in sleep time.

Regarding the age effect of sleep, people under 30 years old had longer baseline sleep and a faster change rate than the other age groups. A reduction in sleep duration with age was reported before, and this could be ascribed to changes in sleep structure and a reduction in acute sleep need [1, 14]. For example, several sleep-related factors were found to decrease with age, including the total sleep time, sleep efficiency, slow-wave sleep and rapid eye movement sleep, while the wake after sleep onset was found to increase with age [25]. Simultaneously, being free of work-related stress, having more leisure time, and taking a nap might augment sleep duration slightly in very old people [4]. Notably, a sharp decrease in sleep duration was observed in the young population, which signalled their risk of insufficient sleep afterwards. The rising popularity of portable electronic devices has been witnessed across the world, and young people have greater access to electronic equipment, while the increasing screen time could induce fast decreases in sleep duration [32]. In addition, nighttime activities and caffeine intake are also important factors related to young persons’ progressively delayed bedtime and short sleep duration [6].

Inconsistent with previous research showing that living in urban areas was associated with poor sleep [6], we found that residence had limited influence on the trajectory of sleep duration. Chinese have witnessed rapid economic growth in the twenty-first century; in particular, the development of logistics supply chains and internet-based technologies provided access to living materials and media entertainment for people living in various places, and the difference between urban and rural areas has been shrinking all the time. Additionally, the baseline BMI category showed no significant influence. Preceding research investigating groups with different BMIs found that their prevalence of short sleep increased to similar degrees [2]. The concurrent influence of BMI on sleep duration requires more complete repeated measurements of BMI in the future. In addition, whether being diagnosed with chronic disease had little influence on the change trajectory and future effort could be done to explore the effects of one specific chronic disease and its severity. Moreover, there was no substantial influence of smoking and drinking status. Smoking behaviour and alcohol involvement were found in both short and long sleepers [33, 34]. Although some smokers experienced disturbed sleep states [11], it was also reported that smokers found cigarettes could help them relax and reduce tension [35]. Poor sleep was found to be associated with heavy alcohol use or addiction [36], but the current analysis was based on whether drink or not; thus, future researchers could collect information about the amount of alcohol consumption and conduct further exploration.

Targeting demographic groups with fast decrease rates is critical since the ability to minimize the negative impact of insufficient sleep is dependent on the identification of key influencing factors so that targeted interventions can be taken. Considering there is a wide range of possibilities that the future sleep duration falls below the standard among individuals with a fast decrease rate, public health efforts should focus on people with steeper change rates, such as females and young individuals. More attention should be devoted to females’ physical and mental health, and appropriate policies should be conducted to improve their working environment and health care system. For the young population, future interventions could target limiting their screen time to prevent insufficient sleep.

The current study went beyond previous studies in several aspects. First, this was the first attempt that explored the change trajectory of sleep duration in a nationally representative population. A longitudinal model was adopted to depict its change pattern and formulate the relationship between sleep duration and time. The results revealed a linear change trajectory with the expected yearly decrease at 2.5 min/day, which could promote a comprehensive understanding of how sleep duration changed over time and provide insights for the prediction and early warning of insufficient sleep. Recognizing the linear change pattern of sleep duration had implications for academic society, which could help deepen the understanding of the change in sleep duration across the lifespan. The systematic development pattern of sleep duration also implied the possibility of investigating the dynamic relationship between sleep duration and other constructs using a multivariate LGM. Moreover, our results presented longitudinal evidence of age and sex differences during the change process of sleep duration and that females and young individuals were prone to a steeper decrease rate, which herald their risk of insufficient sleep. This unique information called for targeted interventions to prevent poor sleep. Meanwhile, there were some covariates whose influence was rather limited (such as residence, initial BMI values, smoking and drinking status), which provided unique reference information for sleep research and interventions in the Chinese context.

Certain limitations should be recognized. Our results were based on subjective reports of sleep duration, and objective measures could be included to provide different perspectives. The findings need to be interpreted with caution because most of the participants were from rural areas and because of the potential bias that might be induced by attrition. Attrition is almost inevitable in longitudinal studies. The attrition rates here were very close to those of other longitudinal research that adopted an open database [31]. Moreover, the present study only explored the influence of initial BMI, and it would be interesting to investigate the relationship between the change in BMI and the change in sleep duration. In addition, other potential confounding variables, such as psychological characteristics, exercise activities, dietary behaviours, and sleep disorders, were not considered here due to low answer rates or incomplete repeated measures, which are worth future examination.

## Conclusion

Our findings formulated the systematic relationship between sleep duration and time, which indicated that the change in sleep duration in Chinese individuals could be depicted by a downward linear trajectory with an expected yearly decrease of 2.5 min/day. The trajectory did not differ by residence, BMI category, chronic disease situation, smoking status, or drinking status. Moreover, there existed sex and age differences in the trajectory, that females and those under 30 were prone to larger decrease rates, and public health interventions should focus on slowing down their decrease rates.

## Data Availability

The datasets used are available from the corresponding author on reasonable request.

## Abbreviations

CHNS: China Health and Nutrition Survey
LGM: Latent growth modelling
BMI: Body mass index
